# Comparable clinical characteristics and outcomes of patients undergoing endovascular treatment for aorto-iliac or femoropopliteal lesions

**DOI:** 10.1007/s12928-025-01143-4

**Published:** 2025-05-24

**Authors:** Yuichi Saito, Yuji Ohno, Kayo Yamamoto, Norikiyo Oka, Masayuki Takahara, Sakuramaru Suzuki, Raita Uchiyama, Masahiro Suzuki, Tadahiro Matsumoto, Yo Iwata, Hideki Kitahara, Yoshio Kobayashi

**Affiliations:** 1 Department of Cardiovascular Medicine, Chiba University Hospital, 1-8-1 Inohana, Chuo-ku, Chiba, Chiba 260-8677 Japan; 2 Department of Cardiology, Japanese Red Cross Narita Hospital, Narita, Japan; 3 Department of Cardiology, Funabashi Municipal Medical Center, Funabashi, Japan; 4 Department of Cardiology, Kimitsu Central Hospital, Kisarazu, Japan; 5 Department of Cardiology, Japan Community Health Organization Chiba Hospital, Chiba, Japan; 6 Department of Cardiology, Chibaken Saiseikai Narashino Hospital, Narashino, Japan

**Keywords:** Peripheral arterial disease, Aorto-iliac, Femoropopliteal, Endovascular treatment

## Abstract

Lower extremity peripheral arterial disease is usually a consequence of advanced atherosclerosis, leading to high mortality and morbidity. Although clinical characteristics and outcomes may differ among patients having peripheral disease in different arterial territories, contemporary data are scarce. From January 2019 to December 2022, this multicenter registry study included 712 patients undergoing endovascular treatment (EVT) for either aorto-iliac (AI) or femoropopliteal (FP) lesions. Patient characteristics and outcomes were compared between AI-EVT and FP-EVT groups. Clinical endpoints included major adverse cardiovascular events, major adverse limb events (MALE), and all-cause mortality, stratified by chronic limb-threatening ischemia (CLTI). Of the 712 patients, 217 (30.5%) and 495 (69.5%) underwent AI-EVT or FP-EVT. Patients undergoing AI-EVT were more likely to be men and current smokers, while diabetes was more frequent in the FP-EVT group. The prevalence of CLTI was significantly higher in the FP-EVT group. In the entire study population, the FP-EVT rather than the AI-EVT group had a significantly higher rate of MALE and mortality, but the incidence of major adverse cardiovascular events was similar between the two groups. When focusing only on patients without CLTI, the mortality risk was similar, while the MALE risk was still higher in the FP-EVT group. In conclusion, patients undergoing EVT for AI and FP lesions in contemporary settings were differently characterized by baseline factors. Although the worse clinical outcomes in the FP-EVT group were mainly driven by the higher prevalence of CLTI, the MALE risk was still increased in patients without CLTI.

**Graphical abstract:**

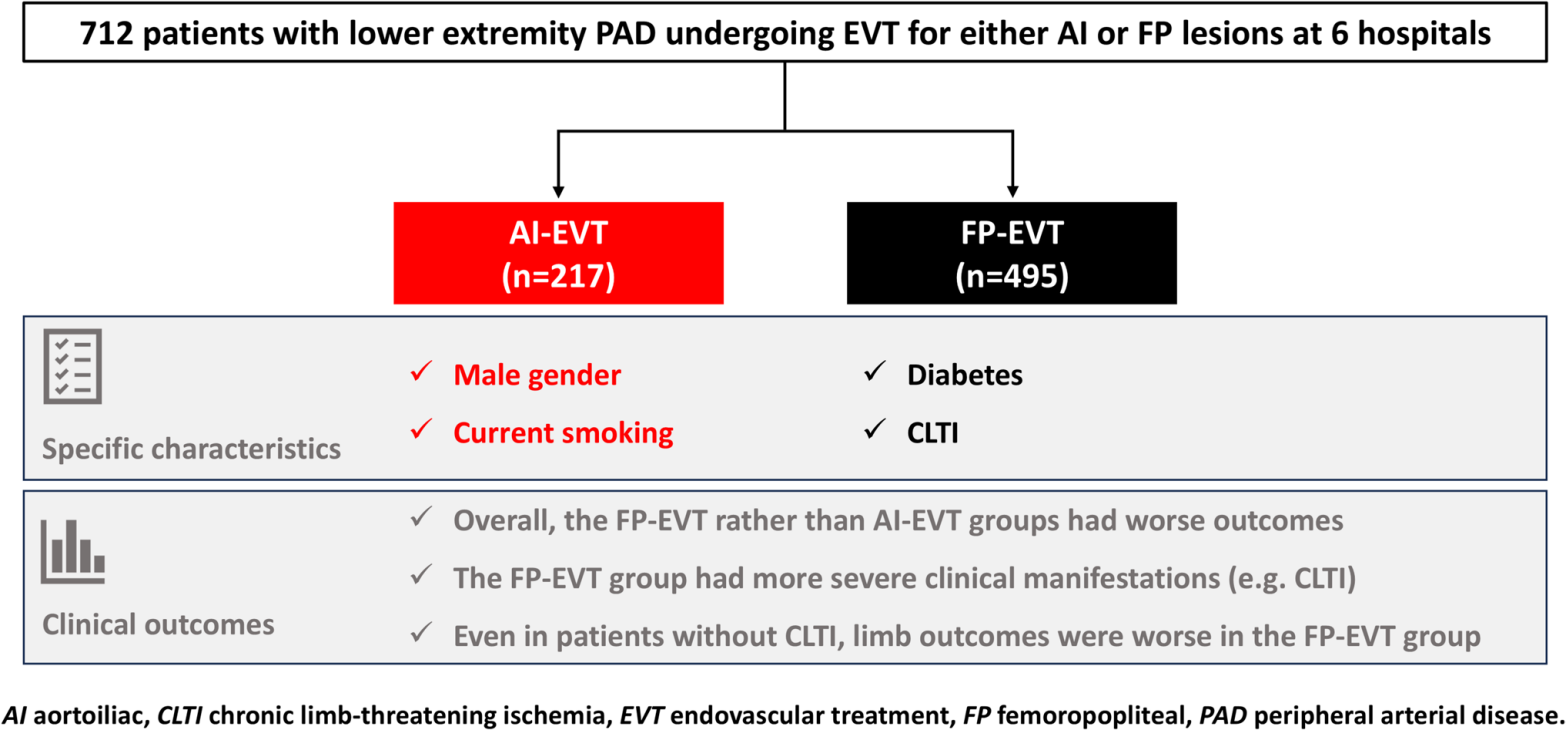

**Supplementary Information:**

The online version contains supplementary material available at 10.1007/s12928-025-01143-4.

## Introduction

Lower extremity peripheral arterial disease (PAD) represents a consequence of advanced atherosclerosis, affecting over 200 million individuals worldwide with an increasing trend in prevalence [1, 2], and the presence of PAD is one of the highest risks for mortality and morbidity in the cardiovascular systems [3, 4]. Patients with lower extremity PAD can have multilevel arterial disease across multiple segments, including aorto-iliac (AI), femoropopliteal (FP), and infrapopliteal lesions [5]. Although endovascular treatment (EVT) is a guideline-endorsed invasive therapeutic option in patients with PAD [5, 6], the indications differ in different arterial segments. For the AI and FP lesions, EVT is recommended when a patient has functionally limiting claudication and inadequate response to medical treatment, while EVT for the infrapopliteal lesions is only considered when chronic limb-threatening ischemia (CLTI) is present [5, 6]. Patients undergoing EVT in the infrapopliteal (below-the-knee) arteries are considerably different from those having AI and FP lesions mainly due to the high prevalence of CLTI, usually requiring dedicated clinical trials for interventional devices in patients having the infrapopliteal lesions [7]. On the other hand, PAD with AI and FP lesions is often grouped together at the levels of guidelines and clinical trials [5, 6, 8, 9]. However, contemporary data are scarce for the comparison between patients with AI and FP lesions [10–12]. Thus, we aimed to evaluate comparable clinical characteristics and outcomes of patients undergoing EVT for the AI and FP lesions in the current real-world practice setting.

## Methods

### Study design and population

**Fig. 1:**
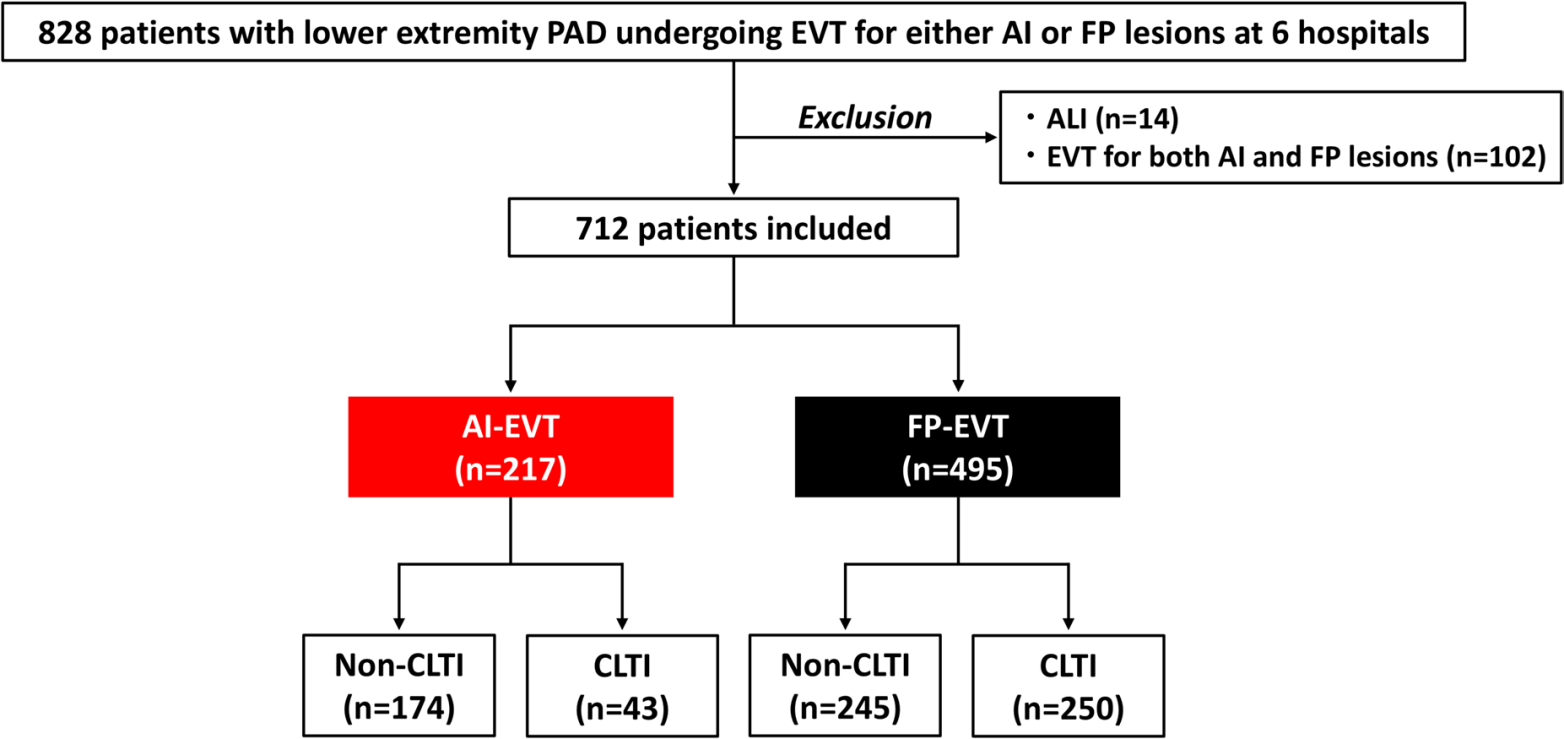
Study flow. *AI* aorto-iliac, *ALI* acute limb ischemia, *CLTI* chronic limb-threatening ischemia, *EVT* endovascular treatment, *FP* femoropopliteal, *PAD* peripheral arterial disease

This was a multicenter, retrospective registry study. From January 2019 to December 2022, a total of 828 patients with lower extremity PAD underwent EVT for AI and FP lesions at six centers in Japan (Funabashi Municipal Medical Center, Kimitsu Central Hospital, Japan Community Healthcare Organization Chiba Hospital, Chiba University Hospital, Narita Red Cross Hospital, and Chibaken Saiseikai Narashino Hospital). PAD was defined by occlusion or stenosis of the lower extremity (AI and FP) arteries on angiography. EVT was indicated for symptomatic PAD (i.e., claudication) (Rutherford category 1–3) or CLTI (Rutherford category 4–6) [5, 6]. All EVT procedures were performed based on the local standard practice, with a bidirectional approach, intravascular ultrasound, drug-eluting stents, drug-coated balloons, and dedicated devices for hemostasis [13, 14]. This study did not include patients undergoing peripheral revascularization only for the infrapopliteal lesions. Patients with acute limb ischemia (*n* = 14) and simultaneous EVT procedures for both AI and FP lesions (*n* = 102) were excluded. Thus, 712 patients with PAD undergoing EVT for either AI or FP lesions were included in this study (Fig. 1). Informed consent for the present study was obtained in an opt-out fashion. This study adhered to the Declaration of Helsinki and received ethical approval from the institutional review committee.

### Definitions and outcomes

Cardiovascular risk factors, such as hypertension, diabetes, dyslipidemia, and current smoking, were defined according to the Japanese Association of Cardiovascular Intervention and Therapeutics criteria [15–17]. Patients were divided into the AI-EVT group and the FP-EVT group (Fig. 1). Data on clinical outcomes events after EVT were ascertained from the medical records at the six centers. The clinical endpoint of the present study included major adverse cardiovascular events (MACE), major adverse limb events (MALE), and all-cause mortality. MACE was a composite of cardiovascular death, myocardial infarction, ischemic stroke, and hospitalization for heart failure, while MALE was defined as a composite of acute limb ischemia, major amputation, and target limb revascularization, according to the consensus documents [5, 18, 19]. Major bleeding events were also evaluated as Bleeding Academic Research Consortium type 3 or 5 [20]. Our main interest was to compare the clinical characteristics and outcomes of patients undergoing AI-EVT and FP-EVT, stratified by the presence of CLTI.

### Statistical analysis

Statistical analysis was performed using EZR (version 1.52; Saitama Medical Center, Jichi Medical University, Saitama, Japan), a graphical user interface for R (The R Foundation for Statistical Computing, Vienna, Austria). All data are expressed as mean ± standard deviation, median [interquartile range], or frequency (percentage). Continuous variables were compared with the Student’s *t* test, while categorical variables were evaluated using Fisher’s exact test. Kaplan–Meier analysis was used to calculate the time to outcome events after EVT, and the log-rank test was employed for between-group comparisons. Multivariate analysis was performed using the Cox proportional hazards models to estimate unadjusted and adjusted hazard ratios with corresponding 95% confidence intervals. Factors with a *p* value < 0.10 in the univariable analysis along with the FP-EVT (versus AI-EVT) were included in the multivariable models. Because of being highly collineated with CLTI, non-ambulatory status was not included in the multivariable analyses with CLTI. A *p* value < 0.05 was considered statistically significant.

## Results

Of the 712 patients, 217 (30.5%) and 495 (69.5%) underwent AI-EVT and FP-EVT, respectively (Fig. 1). Table 1 lists baseline characteristics. Patients in the AI-EVT group were more likely to be men and current smokers, while diabetes was more frequent in the FP-EVT group (Table 1). Of note, the prevalence of CLTI was significantly higher in the FP-EVT group than in the AI-EVT group (Table 1). The baseline characteristics of patients without CLTI are listed in Table S1, showing similar patterns to the entire study population in terms of sex, smoking habits, and diabetes. Table 2 shows the characteristics of EVT procedures. Bare metal stents were the major choice of treatment in the AI-EVT, while drug-coated balloons and drug-eluting stents were predominantly used in the FP-EVT, under the frequent use of intravascular ultrasound in both procedures (Table 2).

**Table 1:** Baseline characteristics

Variable	All (*n* = 712)	AI-EVT (*n* = 217)	FP-EVT (*n* = 495)	*p* value
Age (years)	74.4 ± 8.9	73.8 ± 9.0	74.7 ± 8.8	0.20
Men	517 (72.6%)	187 (86.2%)	330 (66.7%)	< 0.001
Body mass index (kg/m^2^)	22.6 ± 3.6	22.8 ± 3.4	22.6 ± 3.7	0.48
Diabetes	438 (61.5%)	105 (48.4%)	333 (67.3%)	< 0.001
Hypertension	577 (81.0%)	180 (83.0%)	397 (80.2%)	0.41
Dyslipidemia	461 (64.8%)	157 (72.4%)	304 (61.4%)	0.005
Current smoking	127 (17.8%)	63 (29.0%)	64 (12.9%)	< 0.001
Previous CAD	276 (38.8%)	85 (39.2%)	191 (38.6%)	0.93
Previous heart failure	107 (15.0%)	29 (13.4%)	78 (15.8%)	0.43
Atrial fibrillation	113 (15.9%)	30 (13.8%)	83 (16.8%)	0.37
Previous stroke or TIA	112 (15.7%)	28 (12.9%)	84 (17.0%)	0.18
Hemodialysis	141 (19.8%)	28 (12.9%)	113 (22.8%)	0.002
Previous EVT	165 (23.2%)	27 (12.4%)	138 (27.9%)	< 0.001
CLTI	293 (41.2%)	43 (19.8%)	250 (50.5%)	< 0.001
Non-ambulatory status	115 (16.2%)	13 (6.0%)	102 (20.6%)	< 0.001
Hemoglobin (g/dL)	12.4 ± 2.0	12.7 ± 2.0	12.2 ± 2.0	0.002
eGFR (mL/min/1.73 m^2^)	48.7 ± 28.9	55.1 ± 27.0	45.9 ± 29.2	< 0.001
HbA1c (%)	6.6 ± 1.2	6.4 ± 1.1	6.7 ± 1.3	0.02
LDL-C (mg/dL)	93.9 ± 32.0	97 ± 32	93 ± 32	0.14
Medications				
Antithrombotic drugs				
Aspirin	552 (77.5%)	178 (82.0%)	374 (75.6%)	0.06
P2Y12 inhibitors	596 (83.7%)	184 (84.8%)	412 (83.2%)	0.66
Cilostazol	166 (23.3%)	44 (20.3%)	122 (24.7%)	0.21
Oral anticoagulation	137 (19.2%)	37 (17.1%)	100 (20.2%)	0.35
Statin	475 (66.7%)	169 (77.9%)	306 (61.8%)	< 0.001

*AI* aorto-iliac, *CAD* coronary artery disease, *CLTI* chronic limb-threatening ischemia, *eGFR* estimated glomerular filtration rate, *EVT* endovascular treatment, *FP* femoropopliteal, *HbA1c* hemoglobin A1c, *LDL-C* low-density lipoprotein cholesterol, *TIA* transient ischemic attack

**Table 2:** Procedural characteristics

Variable	All (*n* = 712)	AI-EVT (*n* = 217)	FP-EVT (*n* = 495)	*p* value
Chronic total occlusion	279 (39.2%)	66 (30.4%)	213 (43.0%)	0.002
Simultaneous EVT in the BTK arteries	140 (19.7%)	6 (2.8%)	134 (27.1%)	< 0.001
Intravascular ultrasound	558 (78.4%)	193 (88.9%)	365 (73.7%)	< 0.001
Drug-coated balloon	247 (34.7%)	0 (0%)	247 (49.9%)	< 0.001
Bare metal stent	227 (31.9%)	198 (91.2%)	29 (5.9%)	< 0.001
Drug-eluting stent	168 (23.6%)	0 (0%)	168 (33.9%)	< 0.001
Stent graft	19 (2.7%)	0 (0%)	19 (3.8%)	0.002

*AI* aorto-iliac, *BTK* below-the-knee, *EVT* endovascular treatment, *FP* femoropopliteal

**Fig. 2:**
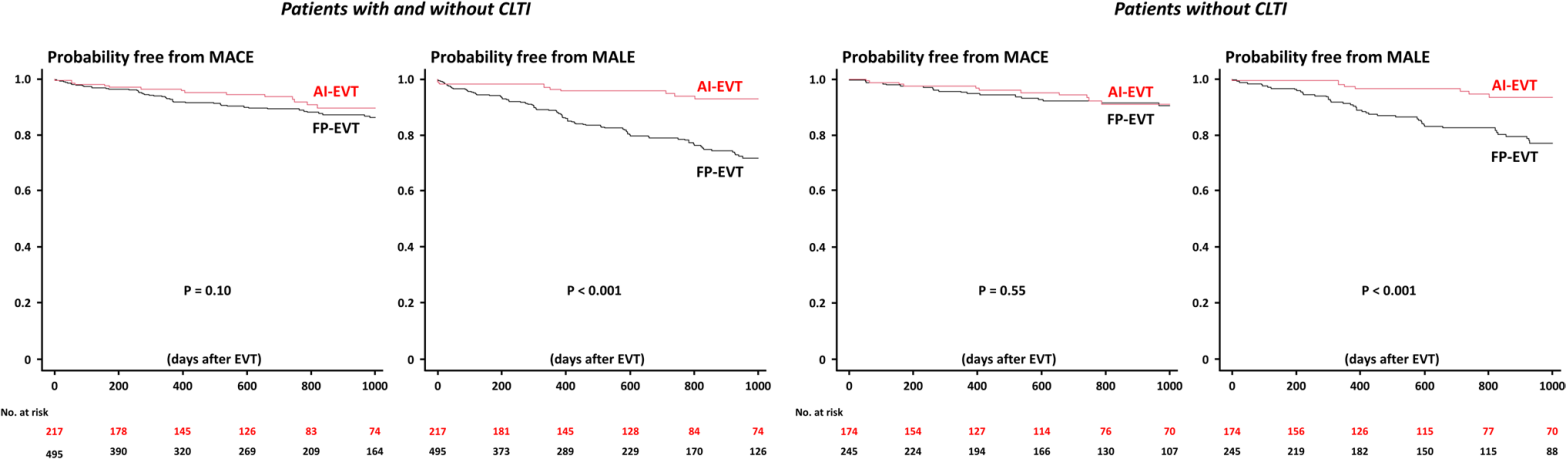
Probability free from MACE and MALE after EVT. *AI* aorto-iliac, *CLTI* chronic limb-threatening ischemia, *EVT* endovascular treatment, *FP* femoropopliteal, *MACE* major adverse cardiovascular events, *MALE* major adverse limb events

**Fig. 3:**
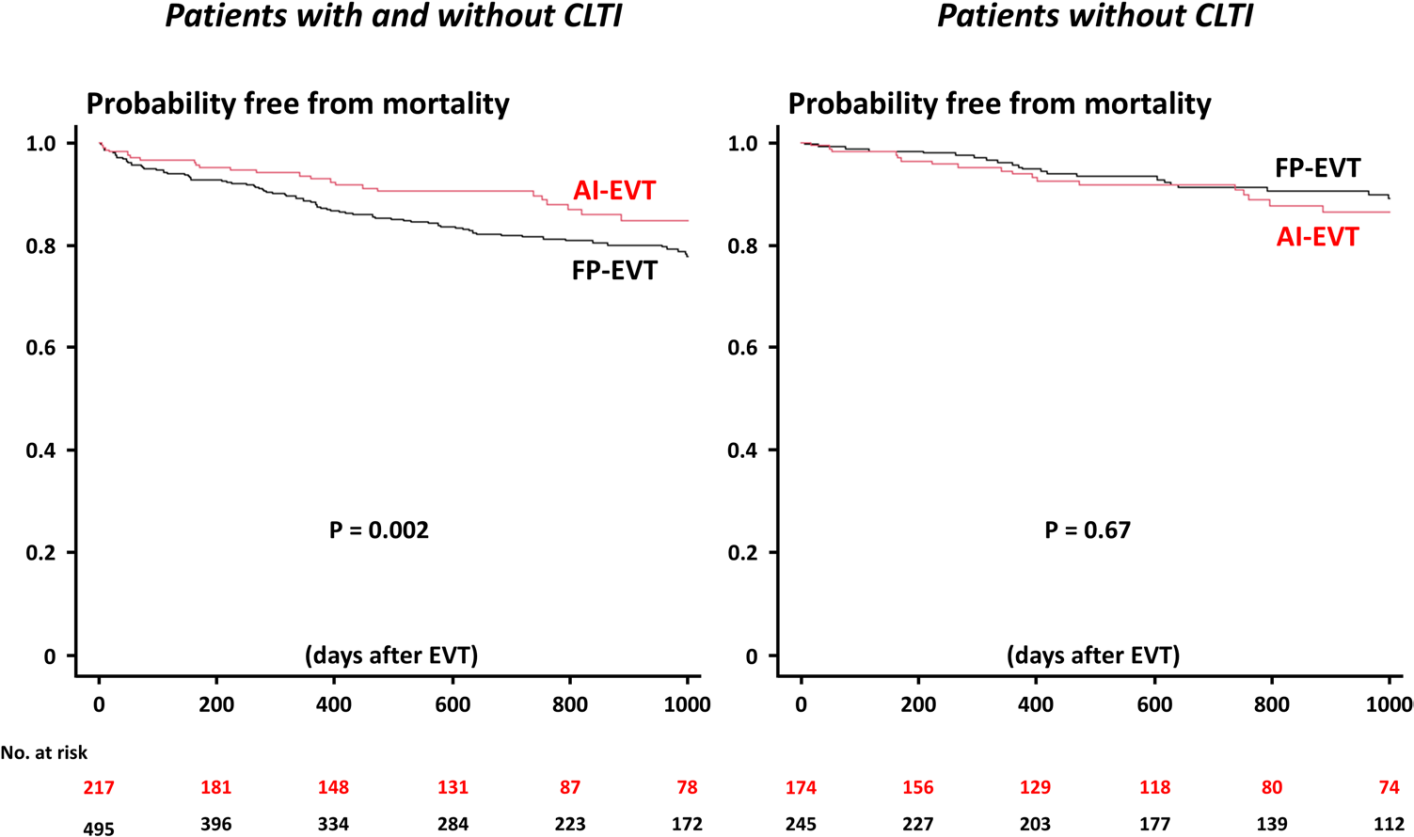
Probability free from all-cause mortality after EVT. *AI* aorto-iliac, *CLTI* chronic limb-threatening ischemia, *EVT* endovascular treatment, *FP* femoropopliteal

During the median follow-up period of 732 [319, 1156] days, 81 (11.4%), 117 (16.4%), and 138 (19.4%) developed MACE, MALE, and all-cause mortality (Table 3). In the entire study population (patients with and without CLTI), Kaplan–Meier analysis demonstrated that the MACE risk was similar between the two groups, while the MALE rates were significantly higher in the FP-EVT group than in the AI-EVT group (Fig. 2), mainly driven by the increased risks of major amputation and target limb revascularization (Table 3). All-cause mortality was also higher in the FP-EVT group (Fig. 3 and Table 3). When focusing only on patients without CLTI, the between-group difference in all-cause mortality was no longer significant (Fig. 3 and Table S2). However, even in patients without CLTI, the FP-EVT group still had a significantly higher risk of MALE (Fig. 2 and Table S2). Univariable and multivariable analyses for MACE, MALE, and all-cause mortality were illustrated in Tables S3, S4, and S5, respectively. Multivariable Cox proportional hazards analysis confirmed the independent prognostic value of FP-EVT (versus AI-EVT) for MALE (Table S4), while no significant effect of AI or FP lesions was found for MACE and all-cause mortality (Table S3, 5). When focusing on patients without previous EVT (*n* = 547), overall results were unchanged (Figure S1).

**Table 3:** Clinical outcomes

Variable	All (*n* = 712)	AI-EVT (*n* = 217)	FP-EVT (*n* = 495)	*p* value
MACE	81 (11.4%)	19 (8.8%)	62 (12.5%)	0.16
Cardiovascular death	26 (3.7%)	5 (2.3%)	21 (4.2%)	0.28
Myocardial infarction	6 (0.8%)	1 (0.5%)	5 (1.0%)	0.30
Ischemic stroke	24 (3.4%)	7 (3.2%)	17 (3.4%)	1.00
Heart failure hospitalization	42 (5.9%)	10 (4.6%)	32 (6.5%)	0.39
MALE	117 (16.4%)	12 (5.5%)	105 (21.2%)	< 0.001
Acute limb ischemia	7 (1.0%)	1 (0.5%)	6 (1.2%)	0.68
Major amputation	25 (3.5%)	3 (1.4%)	22 (4.4%)	0.046
Target limb revascularization	101 (14.2%)	10 (4.6%)	91 (18.4%)	< 0.001
Major bleeding events	29 (4.1%)	8 (3.7%)	21 (4.2%)	0.84
All-cause death	138 (19.4%)	28 (12.9%)	110 (22.2%)	0.004

*MACE* major adverse cardiovascular events, *MALE* major adverse limb events

## Discussion

This multicenter registry study demonstrated that in the current clinical practice in Japan, the number of EVT procedures for the FP lesions was more than double as compared to those for the AI lesions. Patients in the AI-EVT group were characterized by a higher likelihood of male gender and current smoking, while the rates of diabetes and CLTI were higher in the FP-EVT group. Overall, patients in the FP-EVT group had worse clinical outcomes than in the AI-EVT group, mainly driven by severe clinical manifestations represented by CLTI. However, even in patients without CLTI, the FP-EVT rather than the AI-EVT group had worse limb outcomes. These findings confirmed the current clinical patterns of patients with PAD undergoing EVT for AI and FP lesions and suggested that further improvement is needed in EVT for FP lesions.

### Difference across arterial segments

Lower extremity PAD has been recognized as one of the highest-risk populations in cardiovascular diseases. The international REACH registry, which enrolled patients with coronary artery, cerebrovascular, and peripheral artery diseases, showed that PAD was infrequent as compared to coronary artery and cerebrovascular diseases, while the risk of major adverse events (vascular death, myocardial infarction, stroke, and rehospitalization) was higher in the PAD group than the others [3]. However, even within a PAD population, the indication of invasive treatment and clinical outcomes differ across arterial segments. The American guidelines recommend EVT for AI and FP lesions with a Class I indication in patients having significant claudication, while EVT is not indicated for infrapopliteal (below-the-knee) lesions unless CLTI is present [5]. Patients undergoing infrapopliteal EVT inevitably have CLTI, leading to extremely high mortality and morbidities [21]. In the AI lesions, previous reports have shown good long-term limb prognosis after EVT. For instance, a Japanese multicenter observational study demonstrated that 0.9% and 5.7% of patients undergoing EVT for the AI lesions developed major amputation and endovascular or surgical reintervention during the mean follow-up period of 2.6 years [22]. Thus, the 2017 European guidelines endorsed the EVT-first strategy in the AI lesions [23]. On the other hand, despite the recent technological advances including various novel devices (e.g., bare metal and drug-eluting stent, drug-coated balloon, and covered stent), the limb-related outcomes after EVT for the FP lesions remain poor. Even in the contemporary setting, a recent Korean multicenter registry showed that the target lesion revascularization rate two years after EVT for the FP lesions was more than 20% [24]. Although these findings intuitively indicate relatively better clinical outcomes in patients with AI-EVT than in those with FP-EVT, comparable data are scarce in the current practice setting.

### Patient characteristics and outcomes

In the present study, the rates of men and current smokers were higher in the AI-EVT group, while the prevalence of diabetes and CLTI was higher in the FP-EVT group. These findings are in line with previous studies [10, 11]. The differences in the prevalence of cardiovascular risk factors suggest different underlying mechanisms of atherosclerosis in the AI and FP lesions. Despite the contemporary EVT setting represented by the frequent use of intravascular ultrasound and dedicated interventional devices in this study, clinical outcomes were overall poor in the FP-EVT group. One of the important findings of the present study was that there was no significant difference in the incidence of MACE between the two groups. Given the high cardiovascular risk in patients with PAD, the comparative risk of MACE may be of clinical relevance. The mortality risk was higher in the FP-EVT group than in the AI-EVT group in the entire study population, but after excluding patients with CLTI, the difference was no longer significant. The most evident discrepancy in clinical outcomes was an increased risk of target limb revascularization in the FP-EVT group. Notably, CLTI as well as FP-EVT and older age was identified as a factor significantly associated with MALE in the multivariable analysis (Table S4), while diabetes, a well-known predictor of limb revascularization [2], was not. A Japanese multicenter registry study demonstrated that patients with the FP lesions had a two- to three-fold higher risk of MALE after EVT than those with the AI lesions [11]. These findings reinforce the importance of progress in reducing target limb revascularization in the FP-EVT, as was done in the field of coronary intervention [25].

### Study limitations

The present study has some limitations. This was a retrospective study with a moderate sample size. Because the present study aimed to evaluate the comparable characteristics and outcomes of AI- and FP-EVT, patients undergoing peripheral revascularization only for the infrapopliteal lesions were not included. Detailed lesion characteristics (e.g., lesion length, vessel diameter, and calcification on angiography) were not included in the present analysis because of the lack of independent assessment by a core laboratory.

## Conclusions

When comparing patients undergoing AI- and FP-EVT procedures, the AI-EVT group was characterized by a higher likelihood of male gender and current smoking, while diabetes and CLTI were more prevalent in the FP-EVT group. Overall clinical outcomes were poor in the FP-EVT group, which may be due to the severe clinical manifestations including CLTI. However, the risk of MALE, particularly for target limb revascularization, was significantly elevated in the FP-EVT group than in the AI-EVT group, suggesting that there is still much room for improvement in the FP-EVT in indications, pharmacological management, techniques, and technologies.

## Supplementary Information

Below is the link to the electronic supplementary material.

Supplementary file1 Figure S1. Probability free from MACE and MALE in patients without previous EVT. *AI* aorto-iliac, *EVT* endovascular treatment, *FP* femoropopliteal, *MACE* major adverse cardiovascular events, *MALE* major adverse limb events (TIF 632 KB)

Supplementary file2 (DOCX 31 KB)

Supplementary file3 (DOCX 28 KB)

Supplementary file4 (DOCX 29 KB)

Supplementary file5 (DOCX 28 KB)

Supplementary file6 (DOCX 29 KB)

## Data Availability

The identified participant data will not be shared.
