# Supplementary figures and images for: Comparable clinical characteristics and outcomes of patients undergoing endovascular treatment for aorto-iliac or femoropopliteal lesions
Source: Cardiovasc Interv Ther. 2025 May 24;40(4):852–9. doi: 10.1007/s12928-025-01143-4 (PMC12432028; doi:10.1007/s12928-025-01143-4)

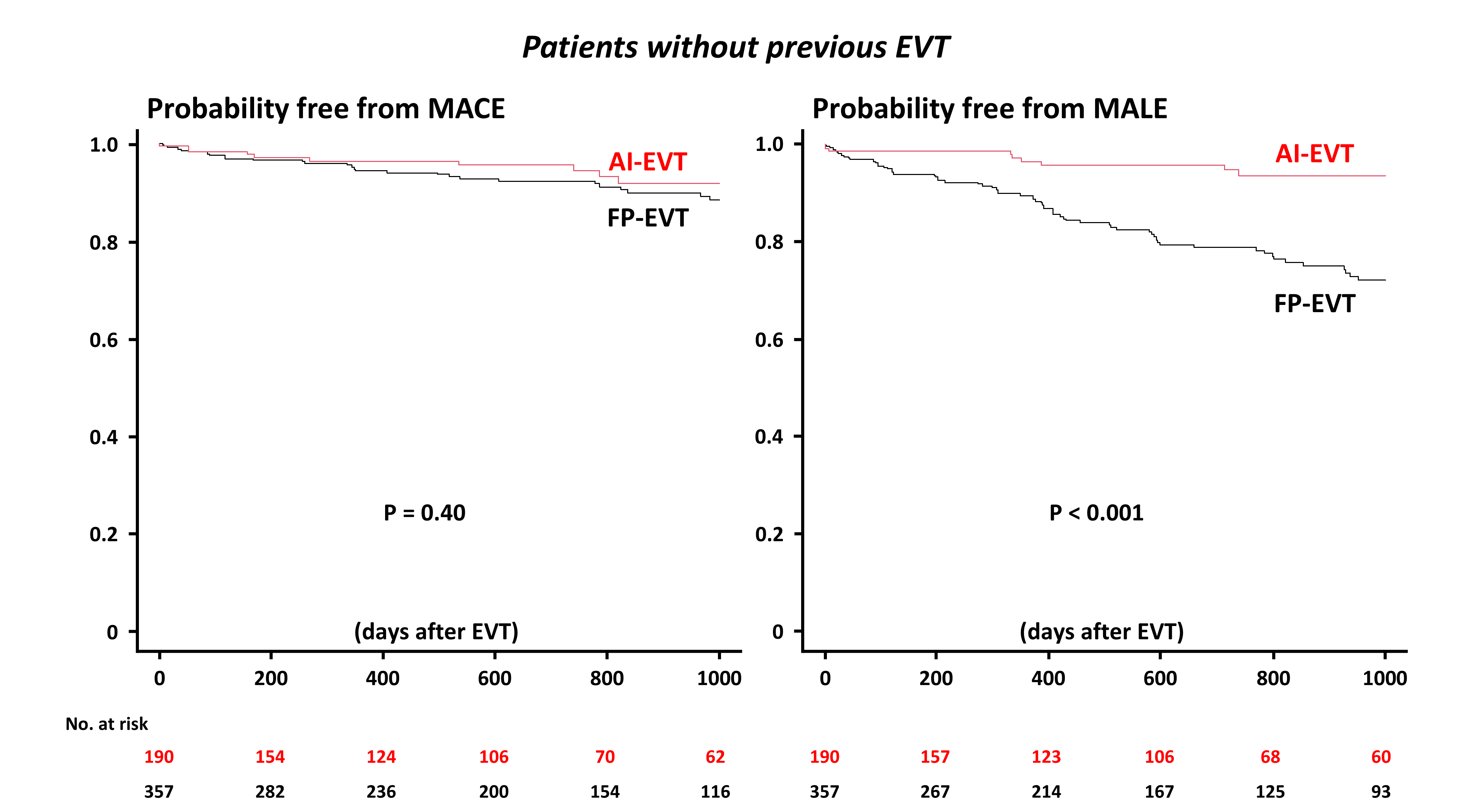

Supplement: Supplementary file 1 — Supplementary file1 Figure S1. Probability free from MACE and MALE in patients without previous EVT. AI aorto-iliac, EVT endovascular treatment, FP femoropopliteal, MACE major adverse cardiovascular events, MALE major adverse limb events (TIF 632 KB) [file 12928_2025_1143_MOESM1_ESM.tif]
