# Supplementary material for: Comparable clinical characteristics and outcomes of patients undergoing endovascular treatment for aorto-iliac or femoropopliteal lesions
Source: Cardiovasc Interv Ther. 2025 May 24;40(4):852–9. doi: 10.1007/s12928-025-01143-4 (PMC12432028; doi:10.1007/s12928-025-01143-4)
Supplement: Supplementary file 2 — Supplementary file2 (DOCX 31 KB) [file 12928_2025_1143_MOESM2_ESM.docx]

**Table S1. Baseline characteristics in patients without CLTI**

| Variable | All  (n=419) | AI-EVT  (n=174) | FP-EVT  (n=245) | P value |
| --- | --- | --- | --- | --- |
| Age (years) | 74.1±8.1 | 73.4±8.6 | 74.5±7.7 | 0.18 |
| Men | 325 (77.6%) | 151 (86.8%) | 174 (71.0%) | <0.001 |
| Body mass index (kg/m^2^) | 23.1±3.4 | 22.9±3.2 | 23.2±3.5 | 0.50 |
| Diabetes | 225 (53.7%) | 76 (43.7%) | 149 (60.8%) | <0.001 |
| Hypertension | 343 (81.9%) | 146 (83.9%) | 197 (80.4%) | 0.37 |
| Dyslipidemia | 303 (72.3%) | 129 (74.1%) | 174 (71.0%) | 0.51 |
| Current smoking | 92 (22.0%) | 52 (30.0%) | 40 (16.3%) | 0.001 |
| Previous CAD | 166 (39.6%) | 68 (39.1%) | 98 (40.0%) | 0.92 |
| Previous heart failure | 48 (11.5%) | 22 (12.6%) | 26 (10.6%) | 0.54 |
| Atrial fibrillation | 50 (11.9%) | 19 (10.9%) | 31 (12.7%) | 0.65 |
| Previous stroke or TIA | 48 (11.5%) | 22 (12.6%) | 26 (10.6%) | 0.54 |
| Hemodialysis | 46 (11.0%) | 17 (9.8%) | 29 (11.8%) | 0.53 |
| Previous EVT | 99 (23.6%) | 26 (14.9%) | 73 (29.8%) | <0.001 |
| Non-ambulatory status | 8 (1.9%) | 2 (1.2%) | 6 (2.5%) | 0.48 |
| Hemoglobin (g/dL) | 12.9±1.9 | 13.0±1.9 | 12.8±1.8 | 0.23 |
| eGFR (mL/min/1.73 m^2^) | 54.2±25.5 | 57.0±25.4 | 52.2±25.5 | 0.06 |
| HbA1c (%) | 6.4±0.9 | 6.3±0.8 | 6.6±1.0 | 0.006 |
| LDL-C (mg/dL) | 95±31 | 96±30 | 95±32 | 0.70 |
| Medications |  |  |  |  |
| Antithrombotic drugs |  |  |  |  |
| Aspirin | 333 (79.5%) | 146 (83.9%) | 187 (76.3%) | 0.07 |
| P2Y12 inhibitors | 358 (85.4%) | 149 (85.6%) | 209 (85.3%) | 1.00 |
| Cilostazol | 113 (27.0%) | 33 (19.0%) | 80 (32.7%) | 0.002 |
| Oral anticoagulation | 71 (17.0%) | 27 (15.5%) | 44 (18.0%) | 0.60 |
| Statin | 308 (73.5%) | 140 (80.5%) | 168 (68.6%) | 0.007 |

*AI* aortoiliac, *CAD* coronary artery disease, *eGFR* estimated glomerular filtration rate, *EVT* endovascular treatment, *FP* femoropopliteal, *HbA1c* hemoglobin A1c, *LDL-C* low-density lipoprotein cholesterol, *TIA* transient ischemic attack.
