# Supplementary material for: Comparable clinical characteristics and outcomes of patients undergoing endovascular treatment for aorto-iliac or femoropopliteal lesions
Source: Cardiovasc Interv Ther. 2025 May 24;40(4):852–9. doi: 10.1007/s12928-025-01143-4 (PMC12432028; doi:10.1007/s12928-025-01143-4)
Supplement: Supplementary file 3 — Supplementary file3 (DOCX 28 KB) [file 12928_2025_1143_MOESM3_ESM.docx]

**Table S2. Clinical outcomes** **in patients without CLTI**

| Variable | All  (n=419) | AI-EVT  (n=174) | FP-EVT  (n=245) | P value |
| --- | --- | --- | --- | --- |
| MACE | 39 (9.3%) | 14 (8.1%) | 25 (10.2%) | 0.50 |
| Cardiovascular death | 9 (2.2%) | 3 (1.7%) | 6 (2.5%) | 0.74 |
| Myocardial infarction | 3 (0.7%) | 1 (0.6%) | 2 (0.8%) | 1.00 |
| Ischemic stroke | 13 (3.1%) | 6 (3.5%) | 7 (2.9%) | 0.78 |
| Heart failure hospitalization | 19 (4.5%) | 7 (4.0%) | 12 (4.9%) | 0.81 |
| MALE | 58 (13.8%) | 9 (5.2%) | 49 (20.0%) | <0.001 |
| Acute limb ischemia | 5 (1.2%) | 1 (0.6%) | 4 (1.6%) | 0.41 |
| Major amputation | 4 (1.0%) | 1 (0.6%) | 3 (1.2%) | 0.64 |
| Target limb revascularization | 55 (13.1%) | 9 (5.2%) | 46 (18.8%) | <0.001 |
| Major bleeding events | 14 (3.3%) | 7 (4.0%) | 7 (2.9%) | 0.59 |
| CLTI | 17 (4.1%) | 4 (2.3%) | 13 (5.3%) | 0.14 |
| All-cause death | 54 (12.9%) | 21 (12.1%) | 33 (13.5%) | 0.77 |

*AI* aortoiliac, *CLTI* chronic limb-threatening ischemia, *EVT* endovascular treatment, *FP* femoropopliteal, *MACE* major adverse cardiovascular events, *MALE* major adverse limb events.
