# Supplementary material for: Comparable clinical characteristics and outcomes of patients undergoing endovascular treatment for aorto-iliac or femoropopliteal lesions
Source: Cardiovasc Interv Ther. 2025 May 24;40(4):852–9. doi: 10.1007/s12928-025-01143-4 (PMC12432028; doi:10.1007/s12928-025-01143-4)
Supplement: Supplementary file 4 — Supplementary file4 (DOCX 29 KB) [file 12928_2025_1143_MOESM4_ESM.docx]

**Table S3. Cox proportional hazards analysis for MACE**

| Variable | Univariable | |  | Multivariable | |
| --- | --- | --- | --- | --- | --- |
|  | HR (95% CI) | P value |  | HR (95% CI) | P value |
| Age (years) | 1.03 (1.00-1.06) | 0.045 |  | 1.02 (0.99-1.06) | 0.21 |
| Men | 0.82 (0.51-1.30) | 0.39 |  |  |  |
| Body mass index (kg/m^2^) | 0.98 (0.93-1.05) | 0.62 |  |  |  |
| Diabetes | 1.52 (0.95-2.42) | 0.08 |  | 0.98 (0.58-1.66) | 0.94 |
| Hypertension | 1.09 (0.61-1.94) | 0.78 |  |  |  |
| Dyslipidemia | 1.06 (0.66-1.70) | 0.81 |  |  |  |
| Current smoking | 0.45 (0.21-0.97) | 0.04 |  | 0.76 (0.34-1.69) | 0.50 |
| Previous CAD | 2.20 (1.42-3.42) | <0.001 |  | 1.74 (1.05-2.89) | 0.03 |
| Previous heart failure | 3.49 (2.22-5.50) | <0.001 |  | 2.00 (1.20-3.33) | 0.007 |
| Atrial fibrillation | 2.47 (1.51-4.03) | <0.001 |  | 1.95 (1.14-3.34) | 0.01 |
| Previous stroke or TIA | 1.51 (0.85-2.69) | 0.16 |  |  |  |
| Hemodialysis | 2.19 (1.34-3.59) | 0.002 |  | 0.97 (0.43-2.16) | 0.94 |
| CLTI | 2.49 (1.60-3.87) | <0.001 |  | 1.66 (1.00-2.78) | 0.052 |
| Non-ambulatory status | 2.24 (1.31-3.85) | 0.003 |  |  |  |
| Hemoglobin (g/dL) | 0.80 (0.71-0.89) | <0.001 |  | 0.93 (0.82-1.04) | 0.20 |
| eGFR (mL/min/1.73 m^2^) | 0.98 (0.97-0.99) | <0.001 |  | 0.99 (0.98-1.01) | 0.21 |
| HbA1c (%) | 0.94 (0.74-1.19) | 0.59 |  |  |  |
| LDL-C (mg/dL) | 1.00 (0.99-1.00) | 0.23 |  |  |  |
| Aspirin | 1.36 (0.77-2.42) | 0.29 |  |  |  |
| P2Y12 inhibitors | 0.87 (0.49-1.54) | 0.62 |  |  |  |
| Cilostazol | 0.72 (0.42-1.25) | 0.24 |  |  |  |
| Oral anticoagulation | 1.13 (0.66-1.96) | 0.66 |  |  |  |
| Statin | 0.93 (0.58-1.47) | 0.74 |  |  |  |
| FP-EVT (vs. AI-EVT) | 1.53 (0.92-2.56) | 0.10 |  | 1.01 (0.58-1.76) | 0.98 |

*AI* aortoiliac, *CAD* coronary artery disease, *CI* confidence interval, *CLTI* chronic limb-threatening ischemia, *eGFR* estimated glomerular filtration rate, *EVT* endovascular treatment, *FP* femoropopliteal, *HbA1c* hemoglobin A1c, *HR* hazard ratio, *LDL-C* low-density lipoprotein cholesterol, *MACE* major adverse cardiovascular events, *TIA* transient ischemic attack.
