# Supplementary material for: Comparable clinical characteristics and outcomes of patients undergoing endovascular treatment for aorto-iliac or femoropopliteal lesions
Source: Cardiovasc Interv Ther. 2025 May 24;40(4):852–9. doi: 10.1007/s12928-025-01143-4 (PMC12432028; doi:10.1007/s12928-025-01143-4)
Supplement: Supplementary file 5 — Supplementary file5 (DOCX 28 KB) [file 12928_2025_1143_MOESM5_ESM.docx]

**Table S4. Cox proportional hazards analysis for MALE**

| Variable | Univariable | |  | Multivariable | |
| --- | --- | --- | --- | --- | --- |
|  | HR (95% CI) | P value |  | HR (95% CI) | P value |
| Age (years) | 0.97 (0.95-0.99) | <0.001 |  | 0.97 (0.95-0.99) | 0.007 |
| Men | 0.74 (0.50-1.09) | 0.12 |  |  |  |
| Body mass index (kg/m^2^) | 0.96 (0.91-1.01) | 0.15 |  |  |  |
| Diabetes | 1.41 (0.96-2.07) | 0.08 |  | 0.99 (0.66-1.51) | 0.98 |
| Hypertension | 0.72 (0.67-1.10) | 0.12 |  |  |  |
| Dyslipidemia | 0.94 (0.64-1.38) | 0.76 |  |  |  |
| Current smoking | 1.06 (0.67-1.69) | 0.81 |  |  |  |
| Previous CAD | 0.89 (0.61-1.30) | 0.54 |  |  |  |
| Previous heart failure | 1.06 (0.63-1.78) | 0.82 |  |  |  |
| Atrial fibrillation | 1.16 (0.70-1.92) | 0.56 |  |  |  |
| Previous stroke or TIA | 1.08 (0.64-1.83) | 0.77 |  |  |  |
| Hemodialysis | 2.18 (1.44-3.30) | <0.001 |  | 1.52 (0.73-3.19) | 0.26 |
| CLTI | 2.50 (1.73-3.62) | <0.001 |  | 1.70 (1.14-2.55) | 0.01 |
| Non-ambulatory status | 1.79 (1.10-2.91) | 0.02 |  |  |  |
| Hemoglobin (g/dL) | 0.94 (0.85-1.03) | 0.17 |  |  |  |
| eGFR (mL/min/1.73 m^2^) | 0.99 (0.99-1.00) | 0.02 |  | 1.00 (0.99-1.01) | 0.79 |
| HbA1c (%) | 1.07 (0.91-1.27) | 0.41 |  |  |  |
| LDL-C (mg/dL) | 1.00 (0.99-1.01) | 0.88 |  |  |  |
| Aspirin | 0.91 (0.59-1.39) | 0.66 |  |  |  |
| P2Y12 inhibitors | 1.06 (0.64-1.78) | 0.82 |  |  |  |
| Cilostazol | 0.83 (0.53-1.28) | 0.39 |  |  |  |
| Oral anticoagulation | 1.13 (0.72-1.79) | 0.60 |  |  |  |
| Statin | 0.80 (0.55-1.17) | 0.24 |  |  |  |
| FP-EVT (vs. AI-EVT) | 4.59 (2.52-8.34) | <0.001 |  | 3.95 (2.13-7.34) | <0.001 |

*AI* aortoiliac, *CAD* coronary artery disease, *CI* confidence interval, *CLTI* chronic limb-threatening ischemia, *eGFR* estimated glomerular filtration rate, *EVT* endovascular treatment; *FP* femoropopliteal, *HbA1c* hemoglobin A1c, *HR* hazard ratio, *LDL-C* low-density lipoprotein cholesterol, *MALE* major adverse limb events, *TIA* transient ischemic attack.
