# Supplementary material for: Comparable clinical characteristics and outcomes of patients undergoing endovascular treatment for aorto-iliac or femoropopliteal lesions
Source: Cardiovasc Interv Ther. 2025 May 24;40(4):852–9. doi: 10.1007/s12928-025-01143-4 (PMC12432028; doi:10.1007/s12928-025-01143-4)
Supplement: Supplementary file 6 — Supplementary file6 (DOCX 29 KB) [file 12928_2025_1143_MOESM6_ESM.docx]

**Table S5. Cox proportional hazards analysis for all-cause mortality**

| Variable | Univariable | |  | Multivariable | |
| --- | --- | --- | --- | --- | --- |
|  | HR (95% CI) | P value |  | HR (95% CI) | P value |
| Age (years) | 1.02 (1.00-1.05) | 0.04 |  | 1.03 (1.00-1.06) | 0.04 |
| Men | 0.94 (0.65-1.35) | 0.72 |  |  |  |
| Body mass index (kg/m^2^) | 0.93 (0.88-0.97) | 0.003 |  | 0.97 (0.92-1.02) | 0.25 |
| Diabetes | 1.42 (0.99-2.03) | 0.055 |  | 1.09 (0.71-1.68) | 0.69 |
| Hypertension | 0.68 (0.46-1.00) | 0.047 |  | 0.51 (0.32-0.80) | 0.004 |
| Dyslipidemia | 0.55 (0.39-0.77) | <0.001 |  | 0.71 (0.47-1.08) | 0.11 |
| Current smoking | 0.51 (0.29-0.90) | 0.02 |  | 0.94 (0.50-1.75) | 0.83 |
| Previous CAD | 1.08 (0.76-1.51) | 0.68 |  |  |  |
| Previous heart failure | 1.91 (1.30-2.82) | 0.001 |  | 0.91 (0.57-1.44) | 0.67 |
| Atrial fibrillation | 2.23 (1.49-3.13) | <0.001 |  | 1.67 (1.06-2.65) | 0.03 |
| Previous stroke or TIA | 1.00 (0.60-1.66) | 0.99 |  |  |  |
| Hemodialysis | 2.83 (1.98-4.05) | <0.001 |  | 0.62 (0.32-1.19) | 0.15 |
| CLTI | 3.49 (2.47-4.93) | <0.001 |  | 2.24 (1.46-3.43) | <0.001 |
| Non-ambulatory status | 3.12 (2.13-4.57) | <0.001 |  |  |  |
| Hemoglobin (g/dL) | 0.74 (0.68-0.81) | <0.001 |  | 0.89 (0.80-0.99) | 0.03 |
| eGFR (mL/min/1.73 m^2^) | 0.98 (0.97-0.98) | <0.001 |  | 0.98 (0.96-0.99) | <0.001 |
| HbA1c (%) | 1.00 (0.84-1.19) | 1.00 |  |  |  |
| LDL-C (mg/dL) | 0.99 (0.99-1.00) | 0.04 |  | 1.00 (0.99-1.00) | 0.30 |
| Aspirin | 0.78 (0.53-1.15) | 0.21 |  |  |  |
| P2Y12 inhibitors | 1.37 (0.81-2.30) | 0.24 |  |  |  |
| Cilostazol | 0.68 (0.44-1.05) | 0.08 |  | 0.77 (0.47-1.27) | 0.30 |
| Oral anticoagulation | 1.24 (0.83-1.87) | 0.29 |  |  |  |
| Statin | 0.59 (0.42-0.83) | 0.002 |  | 0.80 (0.53-1.21) | 0.30 |
| FP-EVT (vs. AI-EVT) | 1.90 (1.25-2.89) | 0.003 |  | 1.18 (0.73-1.92) | 0.50 |

*AI* aortoiliac, *CAD* coronary artery disease, *CI* confidence interval, *CLTI* chronic limb-threatening ischemia, *eGFR* estimated glomerular filtration rate, *EVT* endovascular treatment, *FP* femoropopliteal, *HbA1c* hemoglobin A1c, *HR* hazard ratio, *LDL-C* low-density lipoprotein cholesterol, *TIA* transient ischemic attack.
